# Osteopontin Reduces Biofilm Formation in a Multi-Species Model of Dental Biofilm

**DOI:** 10.1371/journal.pone.0041534

**Published:** 2012-08-07

**Authors:** Sebastian Schlafer, Merete K. Raarup, Peter L. Wejse, Bente Nyvad, Brigitte M. Städler, Duncan S. Sutherland, Henrik Birkedal, Rikke L. Meyer

**Affiliations:** 1 The Interdisciplinary Nanoscience Center (iNANO), Faculty of Science and Technology, Aarhus University, Aarhus, Denmark; 2 Department of Dentistry, Faculty of Health, Aarhus University, Aarhus, Denmark; 3 Department of Bioscience, Faculty of Science and Technology, Aarhus University, Aarhus, Denmark; 4 Stereology and Electron Microscopy Research Laboratory and MIND Center, Aarhus University, Aarhus, Denmark; 5 Arla Foods amba, Viby J., Denmark; 6 Department of Chemistry, Faculty of Science and Technology, Aarhus University, Aarhus, Denmark; Loyola University Medical Center, United States of America

## Abstract

**Background:**

Combating dental biofilm formation is the most effective means for the prevention of caries, one of the most widespread human diseases. Among the chemical supplements to mechanical tooth cleaning procedures, non-bactericidal adjuncts that target the mechanisms of bacterial biofilm formation have gained increasing interest in recent years. Milk proteins, such as lactoferrin, have been shown to interfere with bacterial colonization of saliva-coated surfaces. We here study the effect of bovine milk osteopontin (OPN), a highly phosphorylated whey glycoprotein, on a multispecies *in vitro* model of dental biofilm. While considerable research effort focuses on the interaction of OPN with mammalian cells, there are no data investigating the influence of OPN on bacterial biofilms.

**Methodology/Principal Findings:**

Biofilms consisting of *Streptococcus oralis, Actinomyces naeslundii, Streptococcus mitis, Streptococcus downei* and *Streptococcus sanguinis* were grown in a flow cell system that permitted *in situ* microscopic analysis. Crystal violet staining showed significantly less biofilm formation in the presence of OPN, as compared to biofilms grown without OPN or biofilms grown in the presence of caseinoglycomacropeptide, another phosphorylated milk protein. Confocal microscopy revealed that OPN bound to the surface of bacterial cells and reduced mechanical stability of the biofilms without affecting cell viability. The bacterial composition of the biofilms, determined by fluorescence *in situ* hybridization, changed considerably in the presence of OPN. In particular, colonization of *S. mitis*, the best biofilm former in the model, was reduced dramatically.

**Conclusions/Significance:**

OPN strongly reduces the amount of biofilm formed in a well-defined laboratory model of acidogenic dental biofilm. If a similar effect can be observed *in vivo*, OPN might serve as a valuable adjunct to mechanical tooth cleaning procedures.

## Introduction

Bacteria in dental biofilms produce organic acids upon exposure to fermentable dietary carbohydrates. Repeated pH drops at the biofilmtooth interface lead to slow demineralization of the dental hard tissues and the development of carious lesions. The most common and most effective means of caries prevention is the mechanical removal of dental biofilm. However, self-performed mechanical cleaning using both tooth brush and interdental floss does not result in full removal of the biofilm [1]–[3], and combating the high world-wide prevalence of caries is still one of the major challenges for dental research [4].

A large number of chemical adjuncts to support mechanical tooth cleaning have been developed and proven to contribute to caries control [5]–[7]. While most of these agents, such as chlorhexidine or essential oils, aim at killing bacteria in the oral cavity, non-bactericidal approaches that target bacterial adhesion and biofilm formation have gained increasing attention in recent years [8]–[9]. In particular, milk proteins, such as lactoferrin, α-lactalbumin and caseins have been shown to interfere with the adhesion and biofilm formation of oral organisms [10]–[14]. Since the development of a robust biofilm is a prerequisite for the establishment of highly acidic microenvironments at the tooth surface, these therapeutic approaches possess great potential for caries prevention.

In the present study, we investigate the effect of bovine milk osteopontin (OPN), a glycosylated and highly phosphorylated whey protein, on a well-described laboratory model of dental biofilm composed of *Streptococcus oralis*, *Actinomyces naeslundii*, *Streptococcus mitis*, *Streptococcus downei* and *Streptococcus sanguinis* [15]. OPN is also expressed in a variety of human tissues and involved in numerous biological processes, including bone and tooth mineralization, wound healing and leukocyte recruitment [16]–[18]. While considerable research effort has been spent on describing the interaction of OPN with mammalian cells [19]–[20], no data on the interaction with bacteria and a potential effect of OPN on bacterial biofilm formation have been published. We quantified biofilm formation in a flow cell system spectrophotometrically by crystal violet staining and investigated species composition, cell viability and structural stability of the biofilms by fluorescence labelling and confocal laser scanning microscopy.

## Results and Discussion

**Figure 1:**
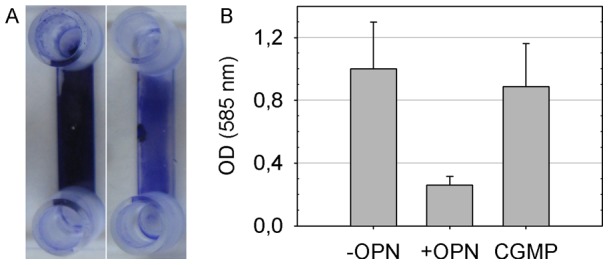
Quantification of biofilm formation by crystal violet staining. Biofilms were grown in flow channels for 30 h on 1/10 diluted THB containing 26.5 µmol/L OPN, 26.5 µmol/L CGMP or none of the two proteins. **A.** Photograph showing biofilms grown with (right channel) and without OPN (left channel) after crystal violet staining. When OPN was present in the medium, less biofilm formed in the flow channels. **B.** Quantification of the biofilm biomass by spectrophotometry. OD_585_ was significantly lower when biofilms were grown in the presence of OPN (+OPN), as compared to biofilms grown on THB only (−OPN). No such effect was observed when CGMP was present in the medium (CGMP). Error bars indicate standard deviations.

Removal of dental biofilm and suppression of biofilm build-up are crucial means of caries prevention. In the five-species model of dental biofilm employed here, bovine milk osteopontin had a profound effect on biofilm growth. When 26.5 µmol/L of OPN were present in the flow medium, biofilm formation in the flow cells was affected considerably (Figure 1A). Quantification of the biofilm biomass by crystal violet staining showed a highly significant difference in OD_585_ between biofilms grown in the absence and presence of OPN (OD_585_ = 1.0±0.30 SD without OPN and 0.26±0.06 SD with OPN; p<0.001). No such effect was observed when biofilms were grown in the presence of caseinoglycomacropeptide (CGMP), another highly phosphorylated milk glycoprotein (0.89±0.27 SD; p = 0.26) (Figure 1B).

**Figure 2:**
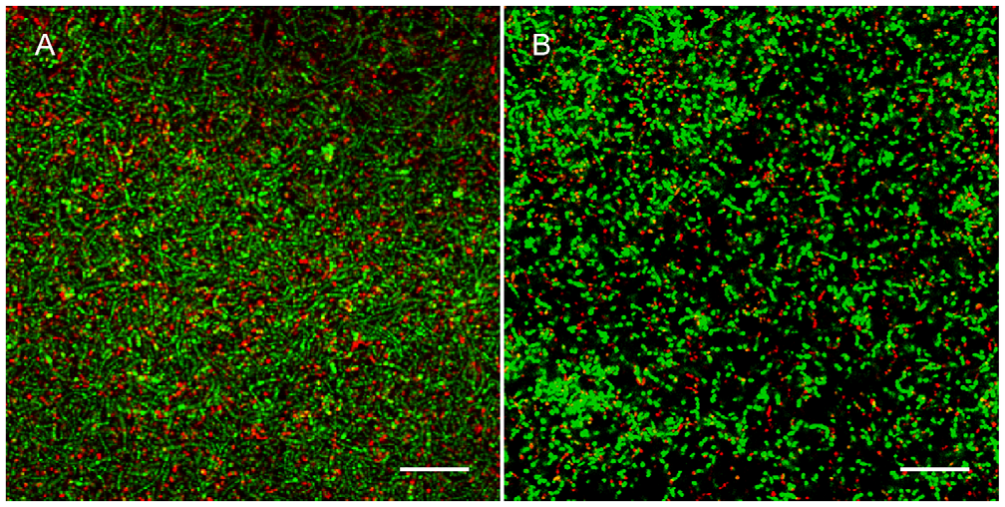
Viability of the organisms in the biofilms. Biofilms grown without OPN (**A**) and with OPN in the medium (**B**) were stained with BacLight. Viable bacteria appear green and membrane-compromised bacteria red. The presence of OPN in the medium did not affect bacterial viability in the biofilms. Bars  = 20 µm.

The observed reduction in biofilm formation could either be caused by a bactericidal effect of OPN, or by an impact on the mechanisms involved in biofilm formation. CGMP and OPN's effect on cell growth was investigated for individual strains in planktonic culture, and none of the two proteins affected the growth of the employed organisms (Figure S1). Metabolic pathways differ considerably between organisms grown in planktonic culture and organisms in biofilms, and we can therefore not exclude that cell division in the biofilms was affected by OPN. Staining with BacLight, however, indicated that most of the bacteria in the biofilms were viable when grown in the presence of OPN (Figure 2).

**Figure 3:**
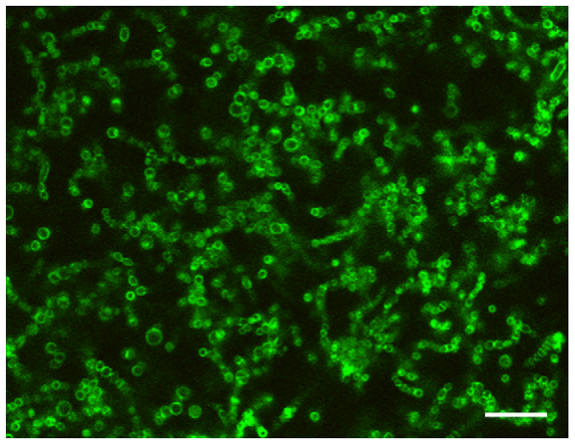
Binding of OPN to bacteria in the biofilms. After growth phase, a biofilm was incubated with fluorescently labelled OPN for 45 min at 35°C. OPN (green) bound to bacterial cell surfaces. Note that chains of streptococci can be recognized, although no bacterial stain was used. Bar  = 10 µm.

This is the first study to investigate the effect of OPN on oral biofilm formation. Some authors have reported that other milk proteins, such as lactoferrin, inhibit initial adhesion of oral organisms to salivary-coated surfaces [11]–[12], but little data have been published on their effect during later stages of biofilm formation. We found that OPN affected biofilm formation, even when added 12 h after initiation of biofilm growth. At 30 h, quantification by crystal violet staining showed a significant difference between control biofilms grown without OPN, and biofilms grown with OPN from 12 h and onwards (OD_585_ = 0.28 (±0.17 SD) with OPN; p<0.001; Figure S2). BacLight staining of 12 h old biofilms showed that the bottom of the flow cell was covered with a monolayer of bacteria, and that patches of multilayered biofilm had started to develop (Figure S3). This suggests that addition of OPN at that time inhibited the further biofilm formation by affecting cell-cell or cell-matrix interactions. Incubation of biofilms with fluorescently labelled OPN showed that the protein adhered to the cell surface of bacteria in the biofilms (Figure 3). When biofilms were grown in the presence of OPN, their stability was compromised and cell mobility increased, as shown by time-lapse imaging of biofilms stained with SYTO9 (Videos S1, S2). In a clinical setting, reduced biofilm stability might facilitate disruption and dislodgement of the biofilms by both professional and self-performed mechanical cleaning procedures.

While biofilm formation was strongly affected by introducing OPN in early stages of growth, the addition of OPN after 28 h did not remove the already established biofilms: No significant difference in biomass quantified by crystal violet staining could be observed (OD_585_ = 1.21±0.35 with OPN, p = 0.26). Collectively, these results show that OPN affects biofilm development, but it does not disperse or disrupt already established biofilms.

**Figure 4:**
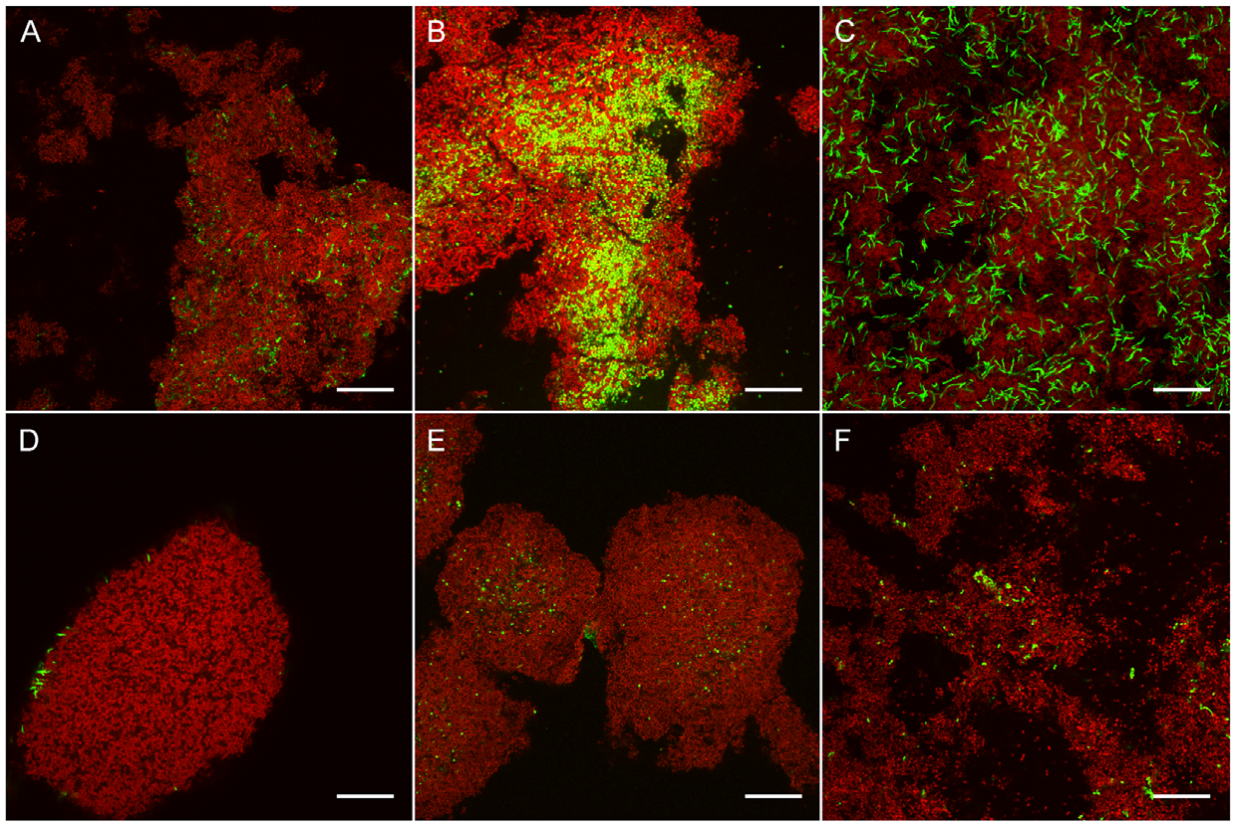
Biofilms grown in the presence of OPN, hybridized with EUB338 and species-specific probes SMIT, SSAN, ANAES, SDOW or SORA2. EUB338 targets all organisms in the biofilms and was labelled with Atto633 (red). Species-specific probes were labelled with Cy3 (green). **A.** *S. mitis* SK24, the dominant organism in biofilms grown without OPN, accounted for 14% of the bacterial biovolume. **B–F.** The relative biovolumes of all other organisms increased in biofilms grown with OPN, as compared to biofilms grown without OPN. *S. sanguinis* SK150 (**B**) was the most abundant organism in the biofilms (48% of the biovolume). *A. naeslundii* AK6 was a prominent colonizer in basal layers of the biofilms (**C**, 22% of the biovolume in the basal layer), but was detected less frequently in upper layers of the biofilm (**D**, 9% of the total biovolume). *S. downei* HG594 (**E**, 11% of the biovolume) and *S. oralis* SK248 (**F**, 3% of the biovolume) represented smaller fractions of the bacterial biofilm.

**Figure 5:**
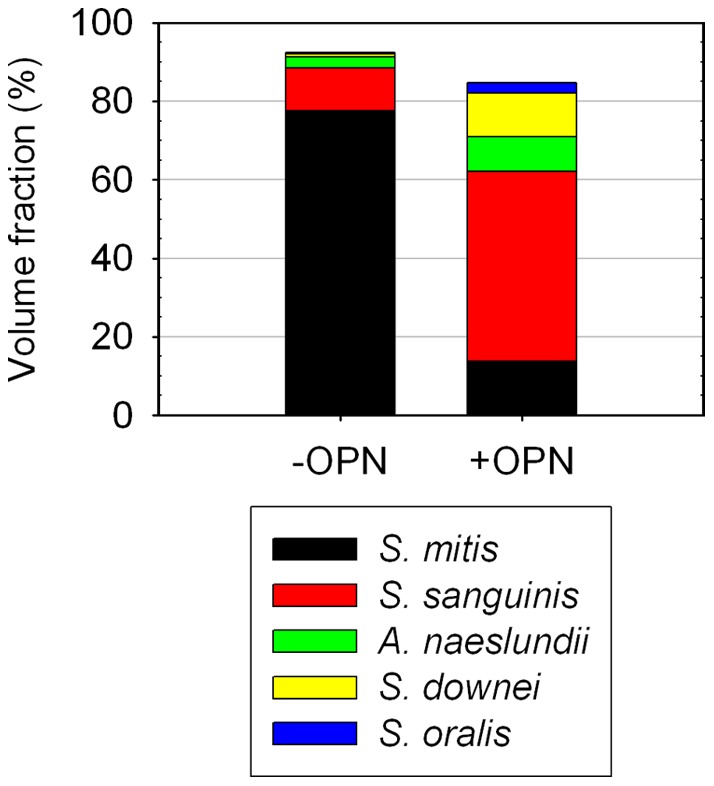
Bacterial composition of biofilms grown in the presence and absence of OPN. In biofilms grown without OPN (−OPN), *S. mitis* SK24 was the predominant organism. When OPN was present in the medium (+OPN), the abundance of *S. mitis* was dramatically lower, and the relative abundance of all other organisms increased. *S. sanguinis* SK150 became the predominant organism.

To further investigate the changes induced by OPN, we subjected biofilms to FISH (fluorescence *in situ* hybridization) with specific probes targeting the five species in the model and determined the bacterial composition. Confocal microscopy and subsequent digital image analysis revealed considerable changes in the absolute and relative biovolumes of individual strains, as compared to biofilms grown without OPN. The abundance of *S. mitis* SK24, the predominant organism in biofilms grown in the absence of OPN, was reduced dramatically from 78% to 14% of the total biovolume (p<0.001). The relative biovolumes of all other organisms increased when OPN was present in the medium (p<0.001), and *S. sanguinis* SK150 became the most abundant organism in the biofilms, representing 48% of the bacterial volume (Figure 4). Detailed biofilm composition data are shown in Figure 5 and Figure S4. The total biovolume detected with EUB338 also decreased significantly (p<0.001), confirming the results obtained by crystal violet staining.

*A. naeslundii* AK6 was inoculated before *S. mitis* SK024 and was predominantly found in the basal layers of the biofilms (Figure 4C, D). As both organisms form coaggregates in OPN-free THB, we hypothesized that attached cells of *A. naeslundii* facilitate the adhesion of *S. mitis*, and that OPN in the medium might interfere with this interaction. However, pairwise coaggregation of planktonic organisms in THB was not affected by the presence of OPN (Table S1). In two-species biofilms, grown with *A. naeslundii* and *S. mitis*, as well as in monospecies biofilms of *S. mitis*, biofilm formation was significantly lower in the presence of OPN (Two-species biofilms: 0.63±0.31 SD without OPN; 0.2±0.11 SD with OPN; p<0.05. Monospecies biofilms: 1.06±0.49 SD without OPN; 0.29±0.12 SD with OPN; p<0.05; Figure S5). While an effect of OPN on receptor-adhesin interactions mediating inter-species coaggregation in the model biofilms cannot be ruled out, these data suggest that OPN also interfered with intra-species coaggregation and bacterium matrix interactions in the biofilms.

Bovine milk osteopontin bound to the bacterial cell surfaces in the employed dental biofilm model. Without affecting cell viability, it reduced biofilm stability and had a highly significant impact on the amount of biofilm formed in the flow cells. If OPN has a similar effect on *in vivo* grown dental biofilms, the protein might be used as a supplement to mechanical tooth cleaning procedures. OPN, provided by, for example, a mouth rinse or a chewing gum during biofilm build-up might compromise dental biofilm stability and reduce the amount of biofilm formed on tooth surfaces. Thereby, the acid challenge would be reduced, and biofilm removal by mechanical debridement might be facilitated. Hence, OPN might be a valuable adjunct to professional and self-performed oral hygiene procedures and contribute to caries control. Further investigations should explore if the results presented here can be extrapolated to *in vivo* grown dental biofilms.

## Materials and Methods

### Bacterial strains

*Streptococcus oralis* SK248, *Streptococcus mitis* SK24, *Streptococcus sanguinis* SK150, *Streptococcus downei* HG594 and *Actinomyces naeslundii* AK6 were used in the experiments. All organisms were moderately acidogenic human oral isolates [15]. 16S rRNA gene sequences have been deposited in GenBank (accession numbers: HQ219654-HQ219658) [21]. All organisms were cultivated aerobically on blood agar (SSI, Copenhagen, Denmark) and transferred to THB (Roth, Karlsruhe, Germany) at 35°C until mid to late exponential phase prior to experimental use.

### Biofilm growth

Biofilms were grown as described previously [15]. Briefly, bacterial cultures (OD = 0.4 at 550 nm) were injected sequentially into flow cells (ibiTreat, µ-slide VI, Ibidi, Munich, Germany) in the following order: 1. *S*. *oralis* SK248; 2. *A*. *naeslundii* AK6; 3. *S*. *mitis* SK24; 4. *S*. *downei* HG594; 5. *S*. *sanguinis* SK150. Each organism was allowed to settle for 30 min, then nonadherent cells were removed by 10 min of flow and the next organism was injected. After inoculation, biofilms were grown for 26 h at 35°C with a flow rate of 250 µl/min (28.3 mm/min), using 1/10 diluted THB (pH 7.0), 1/10 diluted THB containing 26.5 µmol/L (0.9 g/L) OPN (pH 7.0) or 1/10 diluted THB containing 26.5 µmol/L (0.18 g/L) CGMP as the flow medium. OPN and CGMP were added in approximately the same molar concentration. For practical reasons, calculation of the molar concentration of OPN assumed a molecular weight of 34 kDa, although part of the OPN is likely to have formed fractions with lower molecular weight, leading to a slight underestimation of the true molar concentration.

In additional experiments, single species biofilms with *S*. *mitis* SK24 and dual species biofilms with 1. *A*. *naeslundii* AK6 and 2. *S*. *mitis* SK24 were grown in the same way. At least five replicate biofilms were grown for each experimental setting.

### Quantification of biofilm formation

After biofilm growth, THB was removed from the flow channels by aspiration with paper points. The channels were rinsed with distilled water, dried again and stained with 100 µL of 2% crystal violet solution (in 19.2% ethanol containing 0.8% ammonium oxalate) for 1 h. Then channels were rinsed again with distilled water, dried, and filled with 120 µL of absolute ethanol (Sigma-Aldrich, Brøndby, Denmark) for 30 min to destain the biofilms. Thereafter, 100 µL of the stained ethanol solutions, diluted 1∶8, were transferred to a 96 well plate (Sarstedt, Newton, NC, USA), and optical density at 585 nm was measured with a spectrophotometer (BioTek PowerWave XS2, Bad Friedrichshall, Germany). Empty flow channels were processed in the same way and used for background subtraction.

### Growth in planktonic culture

Bacteria were transferred to THB, THB containing 26.5 µmol/L OPN or THB containing 26.5 µmol/L CGMP. Aliquots of 100 µl were transferred to a 96 well plate (Sarstedt, Newton, NC, USA) and OD at 550 nm was measured with a spectrophotometer (BioTek PowerWave XS2, Bad Friedrichshall, Germany). Experiments were carried out in triplicates and repeated once.

### Confocal microscopy

An inverted confocal microscope (Zeiss LSM 510 META, Jena, Germany) equipped with a 63× oil immersion objective, 1.4 numerical aperture (Plan-Apochromat) was used for microscopic analysis unless otherwise stated.

Viability in the biofilms was assessed using BacLight (Invitrogen, Taastrup, Denmark) according to the manufacturer's instructions. 488 nm and 543 nm laser lines were used for excitation. Emission was detected with the META detector set to 500–554 nm and 554–608 nm, respectively. Images were acquired with an XY resolution of 0.4 µm/pixel and a Z resolution corresponding to 2 Airy units (1.6 µm optical slice thickness).

To investigate binding of OPN to the biofilms, the protein was labelled with fluorescein according to the manufacturer's instructions (Invitrogen, Taastrup, Denmark). After growth phase, biofilms were incubated for 45 min with 100 µL of the labelled protein at 35°C and imaged using the 488 nm laser line and a 500–550 nm band pass filter. XY resolution was set to 0.1 µm/pixel and Z resolution corresponded to 1 Airy unit (0.8 µm optical slice thickness).

Biofilm stability was documented by time lapse imaging. Biofilms were stained with SYTO9 (Invitrogen, Taastrup, Denmark), and different microscopic fields of view (20 µm from biofilm substratum interface) were imaged repeatedly for 1000 sec. 488 nm laser line was used for excitation, and emission was detected with the META detector set to 500–554 nm. XY resolution was 0.4 µm/pixel and the Z resolution was set to 2 Airy units (1.6 µm optical slice thickness).

### Biofilm composition analysis

Biofilm composition was determined as described previously [15]. Briefly, biofilms were subjected to FISH with oligonucleotide DNA probes targeting 16S rRNA molecules in bacterial ribosomes. Probe sequences and probe optimization data have been published previously [15]. Each biofilm was hybridized with two probes simultaneously: Probe EUB338, targeting all organisms in the model and one of the five probes SORA2 (specific for *S. oralis*), ANAES (specific for *A. naeslundii*), SMIT (specific for *S. mitis*), SDOW (specific for *S. downei*) and SSAN (specific for *S. sanguinis*). Unlabelled helper probes SORA2H, ANAESH1, ANAESH2, SMITH1, SMITH2, SDOWH and SSANH were employed to enhance the fluorescent signal. All probe sequences have been deposited in Probe Base [22]. Two replicate series of five biofilms were grown and examined independently with confocal microscopy. In each biofilm, 16 fields of view were chosen at random and Z-stacks consisting of six equispaced XY focal planes spanning the height of the biofilms were acquired. The areas of the bacterial mass visualized by EUB338 and the respective species-specific probe were calculated in each image using the program daime [23]. Bacterial biovolumes were estimated for each stack of confocal images by multiplying the area of the bacterial mass with the distance between the layers of the stack [24].

### Coaggregation assays

Bacterial cells were harvested, washed and resuspended in 1/5 diluted THB, 1/5 diluted THB containing 26.5 µmol/L OPN or 1/5 diluted THB containing 26.5 µmol/L CGMP. Suspensions were adjusted to an optical density of 1.0 (550 nm), aliquots of 0.2 mL were mixed and pair coaggregation was evaluated after 30 min, 2 h and 24 h according to the classification of Cisar [25]. Experiments were performed in triplicate and repeated twice.

### Statistical analysis

Unpaired Student's t-tests were employed to assess differences in biofilm growth determined by crystal violet staining. Biofilm composition data was analysed using the Mann–Whitney U test. Absolute and relative biovolumes in the two biological replicates were compared for each strain, and differences in bacterial composition between biofilms grown with OPN and without OPN were analysed. P-values below 0.05 were considered statistically significant.

## Supporting Information

Figure S1 **Effect of OPN and CGMP on bacterial growth in planktonic culture.** **A.** *S. oralis* SK248. **B.** *A. naeslundii* AK6. **C.** *S. mitis* SK24. **D.** *S. downei* HG594. **E.** *S. sanguinis* SK150. Bacterial strains were grown aerobically at 35°C in THB alone (black lines), THB containing OPN (red lines) or THB containing CGMP (green lines). Neither OPN nor CGMP affected planktonic bacterial growth in THB.
(TIF)

Figure S2 **Quantification of biofilm formation by crystal violet staining.** −**OPN**: Biofilms were grown for 30 h on 1/10 diluted THB without OPN. **OPN>12 h**: Biofilm growth was initiated without OPN, and the protein was added to the medium after 12 h. Quantification of the biofilm biomass by crystal violet staining showed a significant effect of OPN on biofilm growth. OD_585_ was significantly lower when OPN was added after 12 h. Error bars indicate standard deviations.
(TIF)

Figure S3 **Biofilm formation after 12 h without OPN.** 12 h old biofilms were stained with BacLight and examined with a wide field microscope (Zeiss Axiovert 200 M, Jena, Germany) equipped with a 100 W high-pressure mercury lamp (HB103, Osram, Winterthur, Switzerland). 12 h after biofilm initiation the bottom of the flow cell was covered with a monolayer of bacteria, and multilayered areas had started to develop. Bar  = 20 µm.
(TIF)

Figure S4 **Detailed biovolume fractions for each organism in biofilms grown with and without OPN.** Each circle represents one microscopic field of view. −**OPN:** Biofilms grown in the absence of OPN. **+OPN:** Biofilms grown in the presence of OPN. Bars indicate means. **A.** *S. oralis* SK248. **B.** *A. naeslundii* AK6. **C.** *S. mitis* SK24. **D.** *S. downei* HG594. **E.** *S. sanguinis* SK150.
(TIF)

Figure S5 **Quantification of biofilm formation by crystal violet staining.** Biofilms were grown for 30 h on 1/10 diluted THB without OPN (−**OPN**) or with OPN (**+OPN**) **A.** Two-species biofilms were grown with *A. naeslundii* and *S. mitis*. **B.** Monospecies biofilms were grown with *S. mitis* alone. For both two-species and monospecies biofilms, OD_585_ was significantly lower when OPN was present in the medium. Error bars indicate standard deviations.
(TIF)

Video S1 **Time-lapse imaging of a biofilm grown without OPN.** After growth phase, the biofilm was stained with SYTO9, and a single field of view (20 µm from the biofilm substratum interface) was imaged for 1000 sec. The biofilm appeared stable and cell mobility was very low. Field of view size: 143×143 µm.
(WMV)

Video S2 **Time-lapse imaging of a biofilm grown in the presence of OPN.** After growth phase, the biofilm was stained with SYTO9, and a single field of view (20 µm from the biofilm substratum interface) was imaged for 1000 sec. Biofilm stability was compromised, and cell mobility was considerably higher than in biofilms grown without OPN. Field of view size: 143×143 µm.
(WMV)

Table S1 **Pairwise bacterial coaggregation.** Pairwise coaggregation was determined 30 min, 2 h and 24 h after mixing in 1/5 diluted THB without OPN (−**OPN**) and with 26.5 µmol/L OPN (**+OPN**). t1 = 30 min; t2 = 2 h; t3 = 24 h. Grade 0: No visible aggregates in cell suspension. Grade 1: Small uniform aggregates in suspension. Grade 2: Definite coaggregates easily seen, but suspension remained turbid without immediate settling of coaggregates. Grade 3: Large coaggregates which settled rapidly leaving some turbidity in the supernatant fluid. Grade 4: Clear supernatant fluid and large coaggregates which settled immediately. No difference in coaggregation patterns was observed between OPN-free THB and THB containing OPN.
(DOC)
