# Supplementary figures and images for: Osteopontin Reduces Biofilm Formation in a Multi-Species Model of Dental Biofilm
Source: PLoS One. 2012 Aug 7;7(8):e41534. doi: 10.1371/journal.pone.0041534 (PMC3413689; doi:10.1371/journal.pone.0041534)

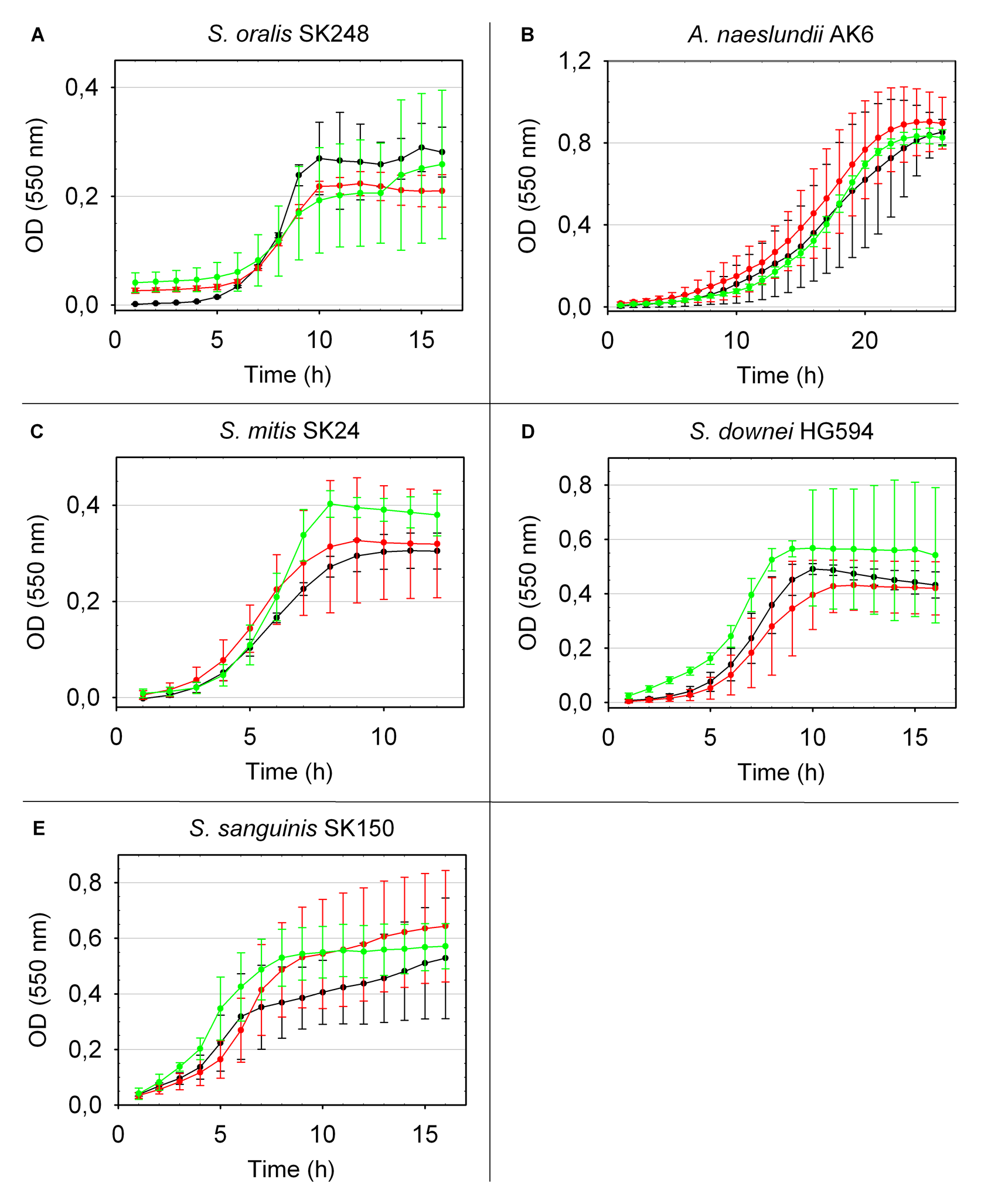

Supplement: Figure S1 — Effect of OPN and CGMP on bacterial growth in planktonic culture. A. S. oralis SK248. B. A. naeslundii AK6. C. S. mitis SK24. D. S. downei HG594. E. S. sanguinis SK150. Bacterial strains were grown aerobically at 35°C in THB alone (black lines), THB containing OPN (red lines) or THB containing CGMP (green lines). Neither OPN nor CGMP affected planktonic bacterial growth in THB. (TIF) [file pone.0041534.s001.tif]

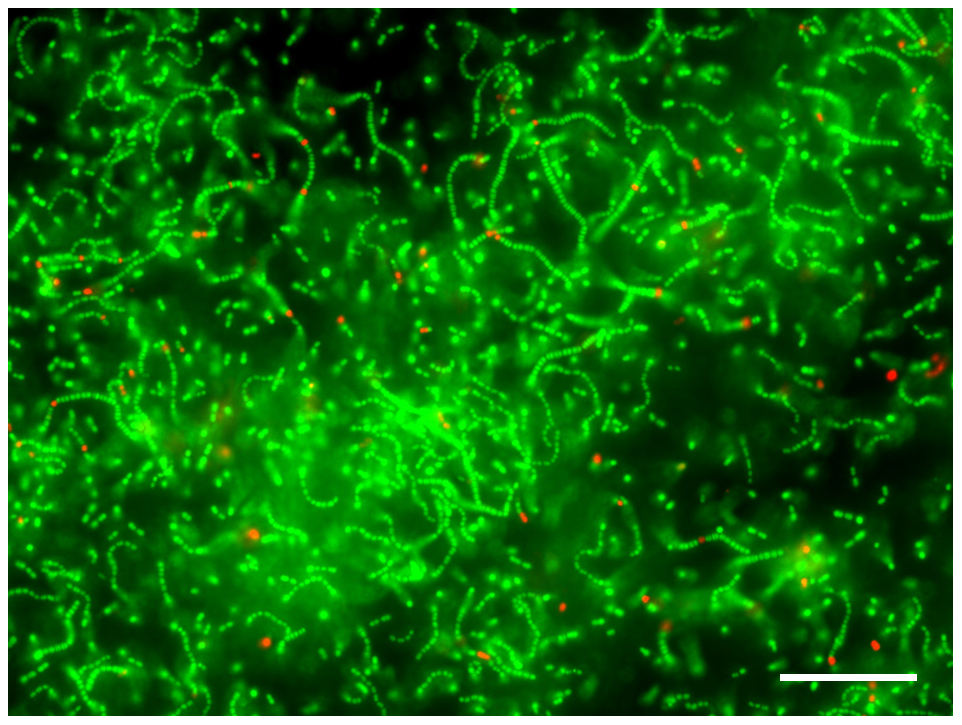

Supplement: Figure S3 — Biofilm formation after 12 h without OPN. 12 h old biofilms were stained with BacLight and examined with a wide field microscope (Zeiss Axiovert 200 M, Jena, Germany) equipped with a 100 W high-pressure mercury lamp (HB103, Osram, Winterthur, Switzerland). 12 h after biofilm initiation the bottom of the flow cell was covered with a monolayer of bacteria, and multilayered areas had started to develop. Bar = 20 µm. (TIF) [file pone.0041534.s003.tif]

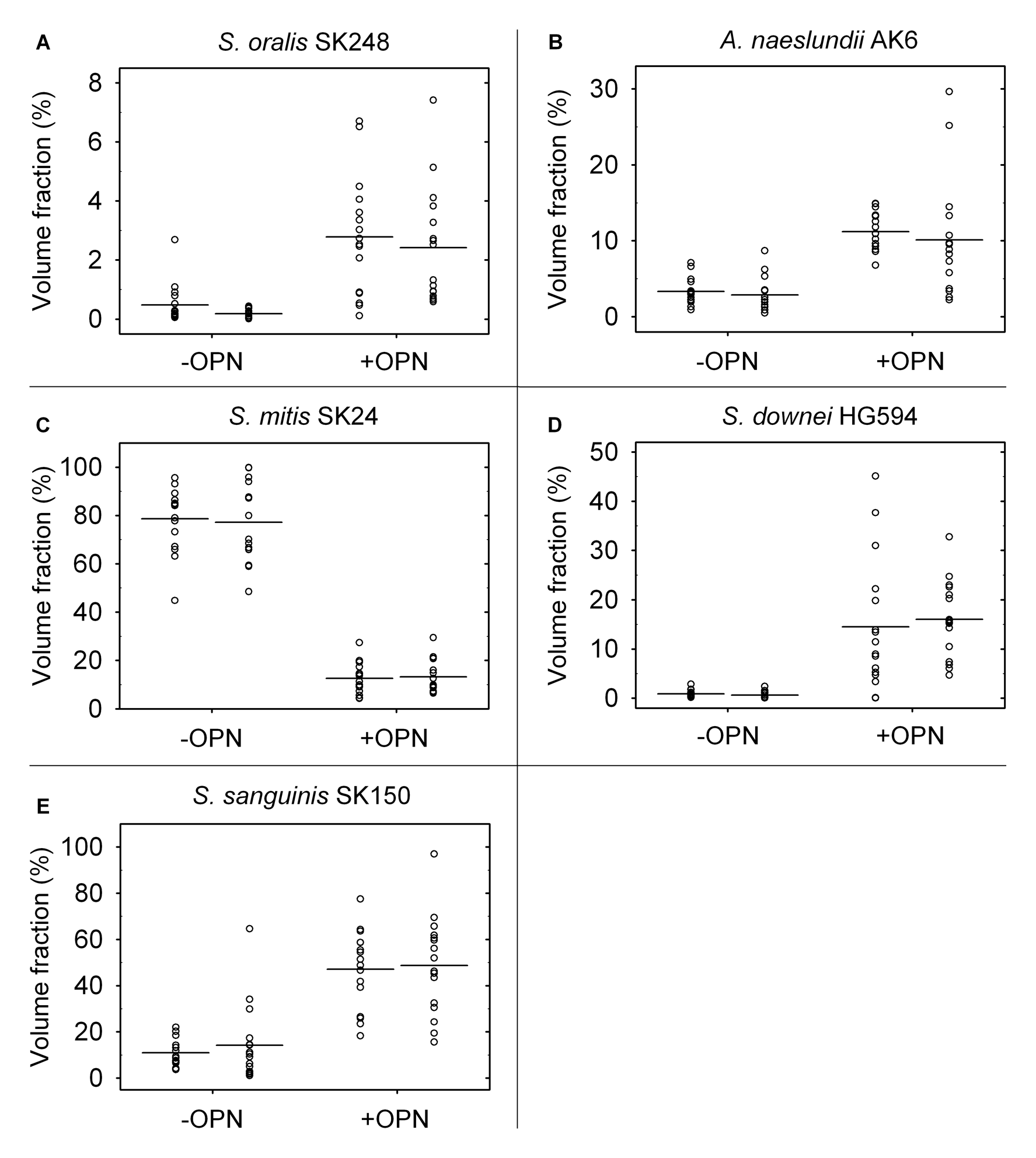

Supplement: Figure S4 — Detailed biovolume fractions for each organism in biofilms grown with and without OPN. Each circle represents one microscopic field of view. −OPN: Biofilms grown in the absence of OPN. +OPN: Biofilms grown in the presence of OPN. Bars indicate means. A. S. oralis SK248. B. A. naeslundii AK6. C. S. mitis SK24. D. S. downei HG594. E. S. sanguinis SK150. (TIF) [file pone.0041534.s004.tif]

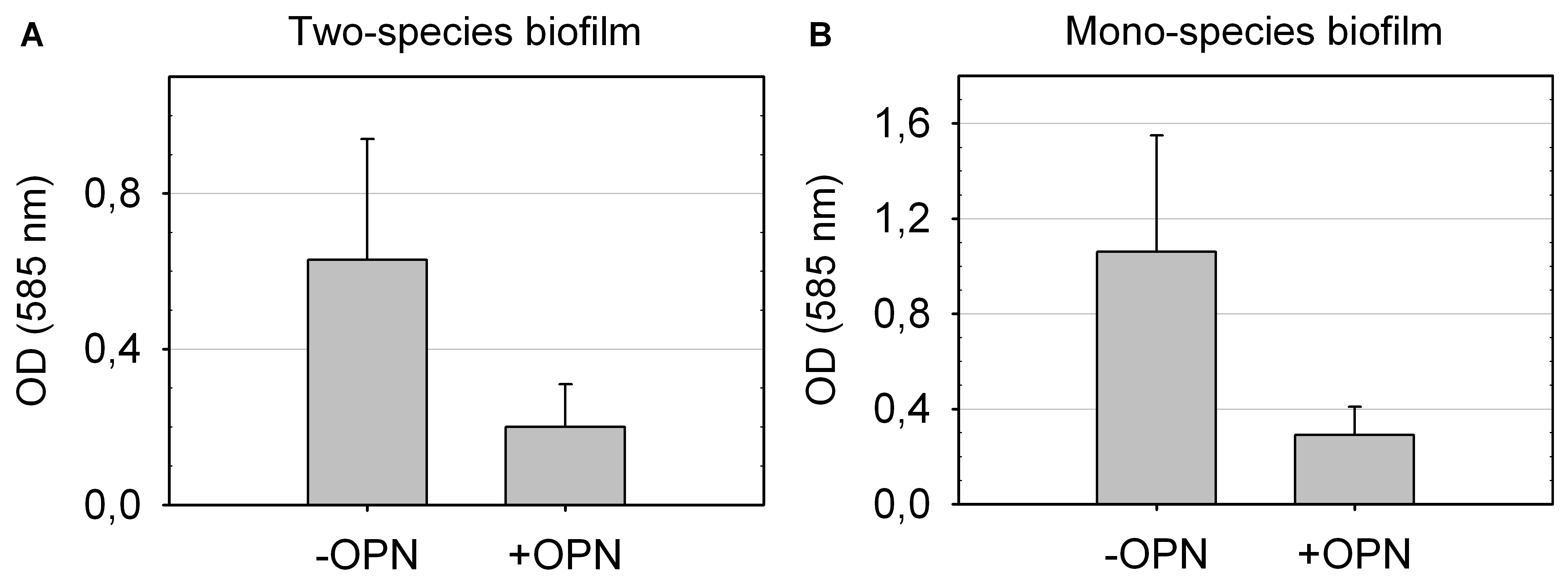

Supplement: Figure S5 — Quantification of biofilm formation by crystal violet staining. Biofilms were grown for 30 h on 1/10 diluted THB without OPN (−OPN) or with OPN (+OPN) A. Two-species biofilms were grown with A. naeslundii and S. mitis. B. Monospecies biofilms were grown with S. mitis alone. For both two-species and monospecies biofilms, OD585 was significantly lower when OPN was present in the medium. Error bars indicate standard deviations. (TIF) [file pone.0041534.s005.tif]
