# Supplementary material for: Osteopontin Reduces Biofilm Formation in a Multi-Species Model of Dental Biofilm
Source: PLoS One. 2012 Aug 7;7(8):e41534. doi: 10.1371/journal.pone.0041534 (PMC3413689; doi:10.1371/journal.pone.0041534)
Supplement: Table S1 — Pairwise bacterial coaggregation. Pairwise coaggregation was determined 30 min, 2 h and 24 h after mixing in 1/5 diluted THB without OPN (−OPN) and with 26.5 µmol/L OPN (+OPN). t1 = 30 min; t2 = 2 h; t3 = 24 h. Grade 0: No visible aggregates in cell suspension. Grade 1: Small uniform aggregates in suspension. Grade 2: Definite coaggregates easily seen, but suspension remained turbid without immediate settling of coaggregates. Grade 3: Large coaggregates which settled rapidly leaving some turbidity in the supernatant fluid. Grade 4: Clear supernatant fluid and large coaggregates which settled immediately. No difference in coaggregation patterns was observed between OPN-free THB and THB containing OPN. (DOC) [file pone.0041534.s008.doc]

**Table S1. Pairwise bacterial coaggregation.**

| **-OPN** | | | | | | | | | | | | |
| --- | --- | --- | --- | --- | --- | --- | --- | --- | --- | --- | --- | --- |
|  | ***S. oralis*** | | | ***S. sanguinis*** | | | ***S. mitis*** | | | ***S. downei*** | | |
|  | t1 | t2 | t3 | t1 | t2 | t3 | t1 | t2 | t3 | t1 | t2 | t3 |
| ***A. naeslundii*** | 0 | 0 | 0 | 0 | 1 | 1 | 2 | 3 | 3 | 0 | 0 | 0 |
| ***S. downei*** | 0 | 0 | 0 | 0 | 0 | 0 | 0 | 0 | 0 | - | - | - |
| ***S. mitis*** | 0 | 0 | 0 | 0 | 0 | 0 | - | - | - | - | - | - |
| ***S. sanguinis*** | 0 | 0 | 0 | - | - | - | - | - | - | - | - | - |
| **+OPN** | | | | | | | | | | | | |
|  | ***S. oralis*** | | | ***S. sanguinis*** | | | ***S. mitis*** | | | ***S. downei*** | | |
|  | t1 | t2 | t3 | t1 | t2 | t3 | t1 | t2 | t3 | t1 | t2 | t3 |
| ***A. naeslundii*** | 0 | 0 | 0 | 0 | 1 | 1 | 2 | 3 | 3 | 0 | 0 | 0 |
| ***S. downei*** | 0 | 0 | 0 | 0 | 0 | 0 | 0 | 0 | 0 | - | - | - |
| ***S. mitis*** | 0 | 0 | 0 | 0 | 0 | 0 | - | - | - | - | - | - |
| ***S. sanguinis*** | 0 | 0 | 0 | - | - | - | - | - | - | - | - | - |

Pairwise coaggregation was determined 30 min, 2 h and 24 h after mixing in 1/5 diluted THB without OPN (**-OPN**) and with 26.5 µmol/L OPN (**+OPN**). t1 = 30 min; t2 = 2 h; t3 = 24 h. Grade 0: No visible aggregates in cell suspension. Grade 1: Small uniform aggregates in suspension. Grade 2: Definite coaggregates easily seen, but suspension remained turbid without immediate settling of coaggregates. Grade 3: Large coaggregates which settled rapidly leaving some turbidity in the supernatant fluid. Grade 4: Clear supernatant fluid and large coaggregates which settled immediately. No difference in coaggregation patterns was observed between OPN-free THB and THB containing OPN.
